# Xylanopectinolytic enzymes by marine actinomycetes from sediments of Sarena Kecil, North Sulawesi: high potential to produce galacturonic acid and xylooligosaccharides from raw biomass

**DOI:** 10.1186/s43141-023-00488-8

**Published:** 2023-03-15

**Authors:** Hana Nadhifah, Nanik Rahmani, Wibowo Mangunwardoyo, Yopi, Akhirta Atikana, Shanti Ratnakomala, Puspita Lisdiyanti

**Affiliations:** 1 Biology Department, Faculty of Mathematics and Natural Sciences, University of Indonesia, Pondok Cina, Depok, West Java 16424 Indonesia; 2 Research Center for Applied Microbiology, Organization Research of Life Sciences and Environment, National Research and Innovation Agency, Jl. Raya Jakarta-Bogor, KM. 46, Cibinong, Bogor, West Java, 16911 Indonesia; 3 Deputy of Regional Research and Innovation Agency, National Research and Innovation Agency, Jl. M.H. Thamrin No.8, Jakarta, 10340 Indonesia; 4 Research Center for Biosystematics and Evolution, Organization Research of Life Sciences and Environment, National Research and Innovation Agency, Jl. Raya Jakarta-Bogor, KM. 46, Cibinong, Bogor, West Java, 16911 Indonesia

**Keywords:** Actinomycetes, Enzyme characterization, Galacturonic acid, Marine sediment, Pectinase, Pectic-oligosaccharides, Xylanase, Xylooligosaccharides

## Abstract

**Background:**

Actinomycetes isolated from marine habitats are known to have the potential for novel enzymes that are beneficial in the industry. In-depth knowledge is necessary given the variety of this bacterial group in Indonesia and the lack of published research. Actinomycetes isolates (BLH 5-14) obtained from marine sediments of Sarena Kecil, Bitung, North Sulawesi, Indonesia, showed an ability to produce pectinase and xylanase that have equal or even higher potential for pectic-oligosaccharides (POS) and xylooligosaccharides (XOS) production from raw biomass than from commercial substrates. This study's objective was to characterize both enzymes to learn more for future research and development.

**Results:**

Pectinase had the highest activity on the 6^th^ day (1.44±0.08 U/mL) at the optimum pH of 8.0 and optimum temperature of 50 °C. Xylanase had the maximum activity on the 6^th^ day (4.33±0.03 U/mL) at optimum pH 6.0 and optimum temperature 60 °C. Hydrolysis and thin layer chromatography also showed that pectinase was able to produce monosaccharides such as galacturonic acid (P1), and xylanase was able to yield oligosaccharides such as xylotriose (X3), xylotetraose (X4), and xylopentaose (X5). BLH 5–14 identified as the genus *Streptomyces* based on the 16S rDNA sequences and the closely related species *Streptomyces tendae* (99,78%).

**Conclusions:**

In the eco-friendly paper bleaching industry, *Streptomyces tendae* has demonstrated the potential to create enzymes with properties that can be active in a wide range of pH levels. The oligosaccharides have the potential as prebiotics or dietary supplements with anti-cancer properties. Further research is needed to optimize the production, purification, and development of the application of pectinase and xylanase enzymes produced by Actinomycetes isolates.

## Background

Oligosaccharides are short carbohydrate polymer chains composed of 2 to 10 monosaccharides. As nutrients for the growth of beneficial microbes in the intestines, oligosaccharides are typically found in the fiber structure of plants and have the potential to serve as prebiotics. Because these oligosaccharides generally cannot be broken down by human enzymes, they can pass through the gut intact. The prevention of harmful bacterial growth, improved mineral absorption, and enhanced gut immunity are a few benefits for humans. Malto-oligosaccharides from starch, fructo-oligosaccharides from sucrose, pectic-oligosaccharides from pectin, and xylo-oligosaccharides from xylan are a few examples of oligosaccharides. These latter two are promising targets for potential prebiotic sources [1, 2].

Oligosaccharides produced by the partial hydrolysis of pectin are known as pectic-oligosaccharides (POS). The hydrolysis process can result in smaller units with different polymerization stages that come from the complex structure of the backbone sides of galacturonic acid and the chain sides of rhamnose and neutral sugars. Because of their anti-cancer, anti-bacterial, and antioxidant characteristics, pectin and POS are utilized in the biomedical sector as dietary fiber and treatments for conditions including ulcers, colon cancer, and diarrhea [3–5]. Research by Wilkowska et al. in 2019 [6] shows the effect of larger-size POS on the growth of the human gut microbiota and inhibition of pathogen growth. POS function as a modulator for immunometabolism in macrophages was studied by Hu et al. in 2021 [7].

On the other side, the hydrolysis reaction of xylan can yield xylo-oligosaccharides (XOS), which are known to be stable in acidic environments. Since 1990, xylo-oligosaccharides have been developed and sold as a food supplement in Japan due to their health benefits, such as anti-tumor and anti-inflammation [8, 9]. One of the newest reports by Abdo et al. in 2021 [10] shows XOS ability to improve gut health in hamsters by reducing plasma cholesterol levels and changing sterols composition. The positive impact of XOS consumption and nutrition as a prebiotic was also demonstrated on human intestinal health through the growth of lactic acid bacteria and *Bifidobacterium* spp. in studies by Lin et al. [11] and Alvarez et al. [12].

The production methodology of oligosaccharides can be chemical by heat and acid treatment or enzymatic, employing pectinase and xylanase enzymes to degrade the product. There has been an investigation regarding pectinase and xylanase enzymes from marine Actinomycetes. Endo-β-1,4-xylanases from *Kitasatospora* sp. and *Streptomyces variabilis* are used in studies by Rahmani et al. [13, 14] to show that they may produce xylooligosaccharides from sugarcane bagasse and beechwood substrate, respectively. Screening results of marine Actinomycetes from Visakhapatnam coast, India, carried out by Yugandhar et al. [15] against 52 isolates, succeeded in obtaining one potential isolate in producing the optimal pectinase enzyme at pH 6.0 media. However, there has not been a research report prior on combined pectinase and xylanase enzymes from marine Actinomycetes isolates. Publications on microorganisms that can produce both enzymes, including the bacteria *Bacillus amyloliquefaciens, Bacillus pumilus, Streptomyces* sp. from terrestrial habitats, and the fungus *Mucor* sp. [16–19].

The lack of research is a significant factor with the increasing need for a mixture of pectinase and xylanase, especially for utilizing waste biomass and industrial base materials containing xylan and pectin substrates. Beyond the benefit of oligosaccharide production, there are many other applications for these two enzymes. The capacity to substitute chemicals in the paper-bleaching process, enhance the extraction and clarification of fruit juices containing hemicellulose and pectin, such as apple and pineapple, and hemp fiber preparation for use in the textile industry are some of these few examples [16, 17, 20–22].

Based on the results of previous research in 2021, screening of 21 Actinomycetes isolates from marine sediments and sponges at Sulawesi and Lampung marine ecosystems in Indonesia. One candidate of Actinomycetes (BLH 5-14) originated from the marine sediments of Sarena Kecil, Bitung City, North Sulawesi, was chosen, and shows potential as a producer of pectinase and xylanase enzymes, with clear zones of 3.6 cm and 3.2 cm on double-layered agar media, respectively. Therefore, further research is needed to explore the potential utilization and characterization of pectinase and xylanase enzymes from Indonesian marine sediments Actinomycetes isolates (BLH 5-14) and their oligosaccharides production using raw biomass.

## Methods

### Microorganism, materials, and chemicals

Actinomycetes (BLH 5-14) are isolated from the marine sediments of Sarena Kecil, Bitung, North Sulawesi, Indonesia. The primary materials used in this research include yeast-malt culture medium with the addition of artificial seawater and pectin from citrus peel [Sigma-Aldrich; St. Louis, MO, USA] or xylan from beechwood [Himedia; Kennett Square, PA, USA] as the glucose substitute. The experiment uses the highest quality and grade of reagents, chemicals, and standards.

### Enzyme production

The enzyme production was carried out based on the method by Rahmani et al. [14], using the marine yeast-malt medium, with the addition of 3% (w/v) Marine ART SF-1, 2% (w/v) commercial pectin substrate from orange peel for pectinase culture, and 2% (w/v) commercial xylan substrate from beechwood for xylanase culture. The production stages consist of the rejuvenation process of BLH 5-14 isolates on a yeast-malt agar medium and cultivation on the 4^th^ day at 28 °C, followed by the pre-culture process on a liquid marine yeast-malt medium that incubates for three days at 28 °C, 190 rpm, and the culture process on liquid marine yeast-malt medium without glucose with the addition of pectin or xylan substrate, that incubates at 28 °C, 190 rpm, for seven days. Samples were taken once every 24 h, and the results were separated by centrifugation at 4 °C for 20 min, 12,000 rpm. The supernatant was stored at 4 °C for further analysis, while the cell pellets were freeze-dried for three days to constant weight.

### Enzyme activity, protein concentration and growth curve

The growth curve was made by measuring the enzyme activity according to the method by Miller [23] and Rahmani et al. [24] on the results of crude enzymes from 0 to 168 h of enzyme production with 3 replications each. The protein concentration during the enzyme production process was carried out according to the BCA Protein Assay Kit [Pierce] protocol, with a standard curve made using bovine serum albumin (BSA) at a concentration of 0.0 - 2.0 mg/mL. The dry weight measurement of pure cells was obtained from freeze-dry results.

### Enzyme activity assay

The enzymatic reaction was based on the method by Rahmani et al. [24], which was carried out by mixing 250 μL of substrate solution with 250 μL of crude enzyme solution at 30 °C for 15 min. Another test tube containing a mixture of 250 μL of the substrate and 250 μL of milli-Q was also reacted as a control at the same time with a blank tube containing 500 μL of the buffer. Dinitrosalicylic acid (DNS) solution of 750 μL was added, and the reaction was heated at 100 °C for 10 min. The reaction tube was then cooled in ice water for 10 min before the optical density (OD) could be measured using a spectrophotometer at a wavelength of 540 nm. The quantity of enzyme needed to produce 1 μmol of reducing sugar every minute under the reaction variables was referred to as an enzyme activity unit (U/mL).

### Enzyme characterization

Enzyme characterization was carried out to calculate optimum pH, optimum temperature, and the influence of metal ions and chemical compounds on enzyme activity. Enzyme activity was measured according to the method by Miller et al. [23]. The combination of pH tested consisted of 50 mM sodium citrate buffer (pH 3.0 - 5.0), 50 mM sodium acetate buffer (pH 4.0 - 6.0), 50 mM sodium phosphate buffer (pH 6.0 - 8.0), 50 mM Tris-HCl buffer (pH 7.0 - 9.0), and 50 mM Glycine-NaOH buffer (pH 8.0 - 10.0). The temperatures were tested from 30 °C to 90 °C. The metal ions solution includes KCl, CaCl_2_, MnCl_2_.4H_2_O, ZnCl_2_, FeSO_4_.7H_2_O, MgSO_4_.7H_2_O_,_ CuSO_4,_ dan HgCl_2_ (5 mM) [Sigma-Aldrich; St. Louis, MO, USA], while the chemical compound includes Triton X-100 [Merck; Jakarta, Indonesia], EDTA [Sigma-Aldrich; St. Louis, MO, USA], PEG-6000 [Merck; Jakarta, Indonesia], methanol [Emsure, ACS; Darmstadt, Germany], ethanol [Emsure, ACS; Darmstadt, Germany], sodium dodecyl sulfate (SDS) [Sigma-Aldrich; St. Louis, MO, USA], and isopropanol [Emsure, ACS; Darmstadt, Germany] (5%).

### Molecular weight analysis with SDS PAGE and zymogram

Running gel and stacking gel were made according to the method of Laemmli [25] and Yopi et al. [26]. Sample preparation consisted of a mixture of 10 μL crude enzyme and 10 μL loading buffer. SDS PAGE and zymogram settings are 200 W, 120 V, and 50 mA for 100 min. SDS PAGE staining using coomassie brilliant blue G250 solution for one night and de-staining for 1 h. Staining of the zymogram was carried out in stages using Triton X-100 (2.5%), milli-Q, incubation in a 50 mM buffer with optimum pH and optimum temperature, Lugol dye for pectin and congo red dye for xylan, and de-staining with 1M NaCl solution and 0.5% (v/v) acetic acid solution.

### Thin layer chromatography on hydrolysis products of pectinase and xylanase

The hydrolysis reaction was done based on the method by Rahmani et al. [27] by mixing 1% (w/v) pectin and xylan substrates in a 50 mM buffer at optimum pH with crude pectinase and xylanase enzymes (1:1). The substrates used for pectinase include commercial substrates of pectin from apples and orange peels, as well as biomass substrates of apple peels, orange peels, and cacao peel. The substrates used for xylanase include commercial substrates of xylan from beechwood, bagasse biomass, palm kernel cake, and xylan extracts from corn cobs and tobacco plants. The entire substrate mixture was then incubated at 40 °C with reaction sampling carried out at 0, 1, 4, 24, 48, and 72 h. The samples were heated at 90 °C for 10 min. The results were separated by centrifugation for 20 min at 4 °C, 12,000 rpm. Thin layer chromatography (TLC) was performed on silica gel paper (20x10 cm) with a mixture of butanol, acetic acid glacial, and milli-Q (2:1:1) as the liquid phase, and diphenylamine acetone phosphoric acid (DAP) as spray solution.

### Molecular identification of isolate BLH 5-14

The DNA extraction steps were carried out according to the protocol on the Wizard Genomic DNA Purification Kit [Promega]. The DNA extraction results then entered the PCR stage using the EmeraldAmp GT PCR Master Mix with a total volume of 100 μL. The primers used were 9F (5’ AGRGTTTGATCMTGGCTCAG 3’) and 1510R (5’ TACGGYTACCTTGTTAYGACTT 3’) with cycles according to the method by Hayat et al. [28]. The sequencing process was carried out with the assistance of the Apical Scientific Laboratory, Selangor, Malaysia, and mediated by PT. Genetics Science, Tangerang, Banten, Indonesia. The sequences were then analyzed using the FinchTV application, DNA Baser Assembler, Bioedit, and NCBI DNA Blast. The phylogenetic tree was created using MEGA X.

## Results

### Enzyme production, protein concentration and growth curve

**Fig. 1:**
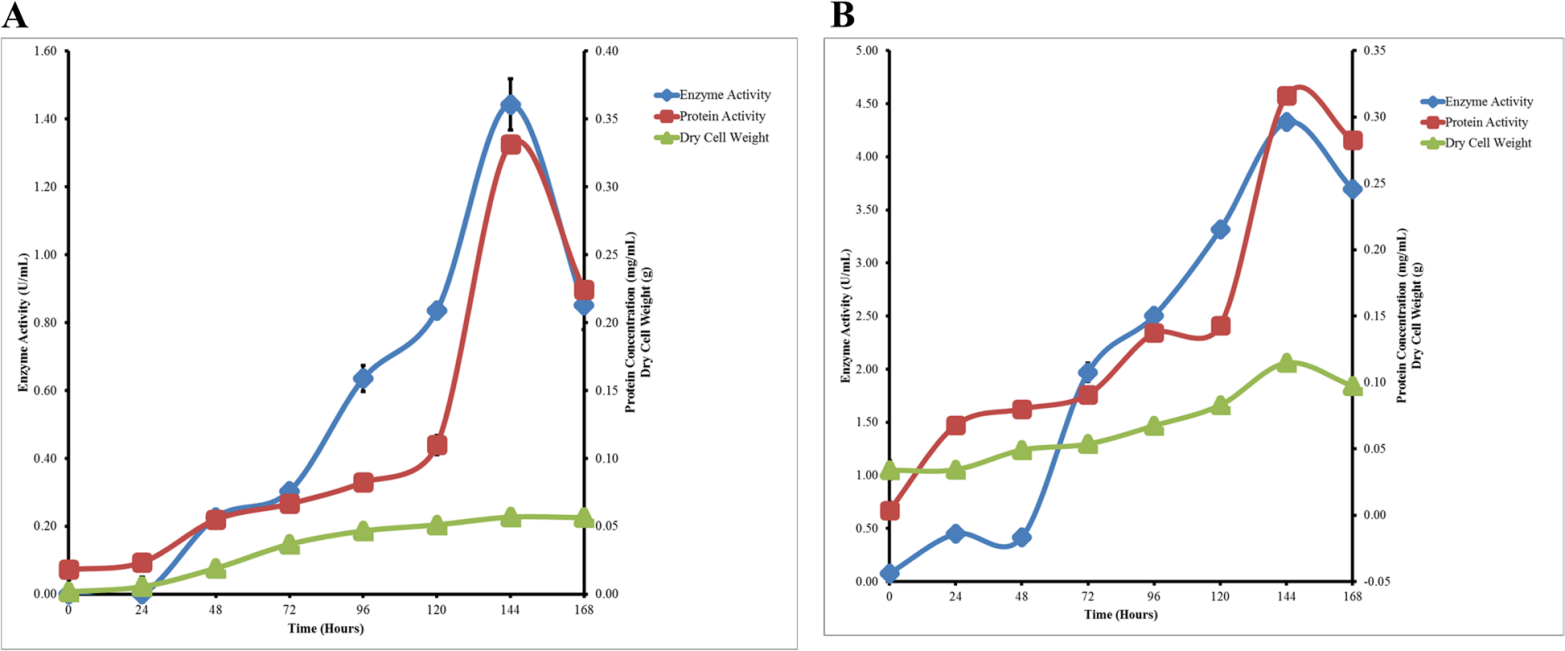
Production curve of pectinase (**A**) and xylanase (**B**) for 7 days culture production. Sampling was done every 24 h. Enzyme activity, protein concentration, and dry cell weight was calculated

Fig. 1 shows that enzyme activity, protein concentration, and total dry weight of cells in pectinase and xylanase production cultures continued to increase until day 6^th^ and decreased on day 7^th^. The highest pectinase enzyme activity was 1.44±0.08 U/mL, the protein was 0.33 mg/mL, and dry cell weight was 0.0567 g, while the optimum xylanase enzyme activity was 4.33±0.03 U/mL, protein of 0.32 mg/mL, and dry cell weight of 0.1147 g.

### Enzyme characterization

**Fig. 2:**
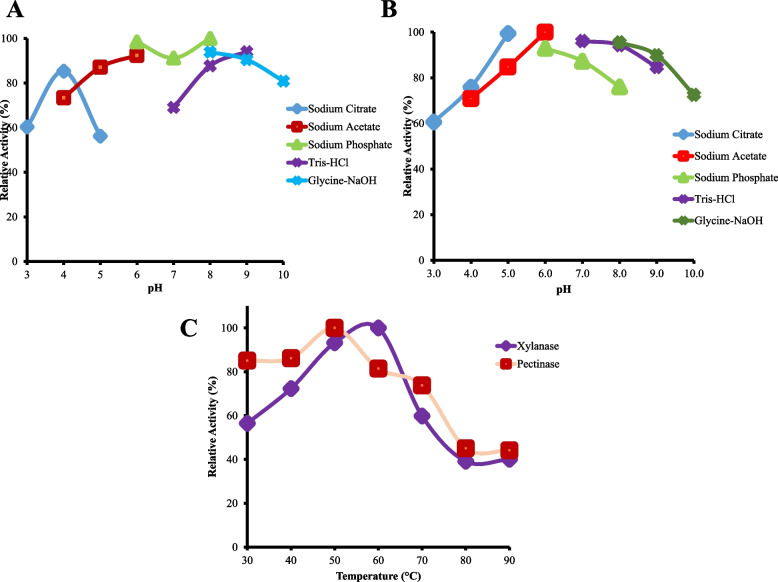
Effect of different buffers, pH (**A-B**), and temperatures (**C**) on pectinase and xylanase activity. **A** Effect of buffers and pH on pectinase. **B** Effect of buffers and pH on xylanase. The reaction for optimum pH characterization was done under the same condition reactions at 30°C for 15 min, while the reaction for optimum temperature characterization was done using optimum pH for 15 min reaction time

The results of enzyme characterization for optimum pH of pectinase and xylanase are in Fig. 2A and B. The pectinase enzyme showed the highest activity in sodium phosphate buffer pH 8.0 at 5.08±0.17 U/mL, and xylanase enzyme showed the highest activity at sodium acetate buffer pH 6.0 at 3.58±0.01 U/mL. The activity of the pectinase enzyme in Fig. 2C increased to a temperature of 50 °C by 5.08±0.17 U/mL and then decreased to a temperature of 90 °C. At the same time, the activity of the xylanase enzyme still increased to a temperature of 60 °C by 6.22±0.04 U/mL.

The results in Table 1 show that pectinase and xylanase have a drastic decrease in activity by Hg^2+^ ions with activity values of 0.00±0.08 U/mL (0%) and 0.65±0.13 U/mL (11%), respectively. On the other hand, the addition of K^+^, Mn^2+^, and Fe^2+^ ions result in increased activity. The pectinase enzyme activity values for K^+^, Mn^2+^, and Fe^2+^ ions were 6.70±0.01 U/mL (132%), 7.57±0.27 U/mL (149%), and 10.62±0.09 U/mL (209%), while in xylanase it was 8.39±0.12 U/mL (135%), 13.62±0.04 U/mL (219%), and 13.38±0.14 U/mL (215%), consecutively. Characterization of chemical compounds showed the highest activity inhibition by SDS for both enzymes, followed by isopropanol, methanol, and ethanol.

**Table 1:** Influence of metal ions and chemical compounds on pectinase and xylanase enzymes

Metal ions/chemical	Relative activity (%)	
	Pectinase	Xylanase
Control	100±0.17	100±0.04
KCl	132±0.01	135±0.12
CaCl_2_	83±0.22	140±0.11
MnCl_2_.4H_2_O	149±0.27	219±0.04
ZnCl_2_	161±0.03	137±0.09
Fe_2_SO_4_.7H_2_O	209±0.09	215±0.14
MgSO_4_.7H_2_O	80±0.30	144±0.04
CuSO_4_	73±0.03	35±0.03
HgCl_2_	0±0.08	11±0.13
PEG-6000	83±0.05	130±0.07
Isopropanol	63±0.02	106±0.02
Methanol	56±0.02	104±0.03
Ethanol	52±0.01	99±0.02
Triton X-100	83±0.03	133±0.36
EDTA	106±0.02	60±0.04
SDS	0±0.00	0±0.01

### Enzyme molecular weight analysis

**Fig. 3:**
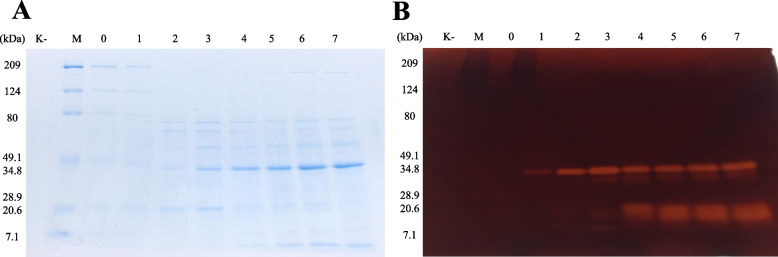
SDS PAGE (**A**) and zymogram (**B**) of xylanase crude enzyme. M, molecular weight marker; lane 0-7: culture supernatant sampling of enzyme production day 0-7; K-, negative control

SDS PAGE and zymogram analysis on the pectinase enzyme did not produce clear enzyme bands. In contrast, the SDS PAGE and zymogram on the xylanase enzyme in Fig. 3 show the separation of enzyme bands from xylanase culture samples, with the size between 34.8 and 25 kDa, respectively.

### Thin layer chromatography hydrolysis product analysis

**Fig. 4:**
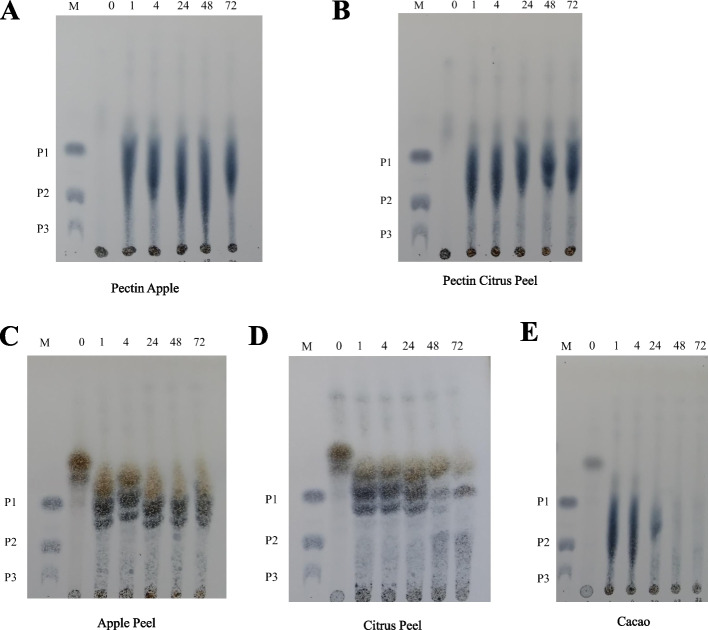
TLC Analysis of hydrolysis products from various pectin commercial substrates and biomass (**A-E**). **A** Pectin from apple. **B** Pectin from citrus peel. **C** Biomass apple peel. **D** Biomass citrus peel. **E** Biomass cacao; M, Standards; P1, Galacturonic acid; P2, Digalacturonic acid; P3, Trigalacturonic acid

**Fig. 5:**
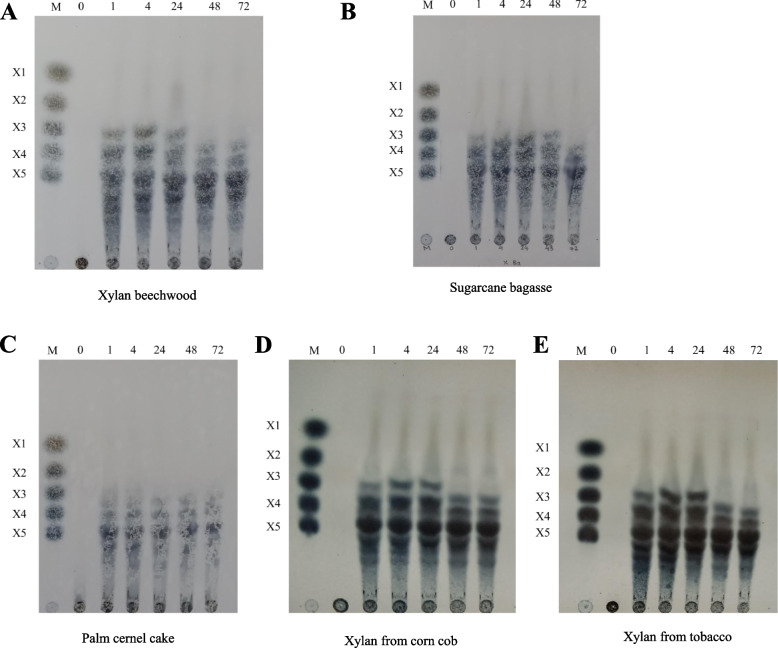
TLC Analysis of hydrolysis products from various xylan commercial substrates and biomass (**A-E**). **A** Xylan from Beechwood. **B** Bagasse. **C** Palm kernel cake. **D** Corn Cob. **E** Tobacco; M, Standards; X1, Xylose; X2, Xylobiose; X3, Xylotriose; X4, Xylotetraose; X5, Xylopentaose

Based on Fig. 4 shows the oligosaccharide product of pectinase in the form of galacturonic acid (P1) from the biomass of apple peels and orange peels from the 1^st^ hour. Fig. 5 shows the results of oligosaccharide products of xylanase in the form of xylotriose (X3), xylotetraose (X4), and xylopentaose (X5) on all substrates from the 1^st^ hour, with xylotriose starting to become depleted at 48 h. Production of XOS can be seen similarly between raw biomass and commercial substrates of beechwood xylan.

### 16S rDNA Molecular identification

**Fig. 6:**
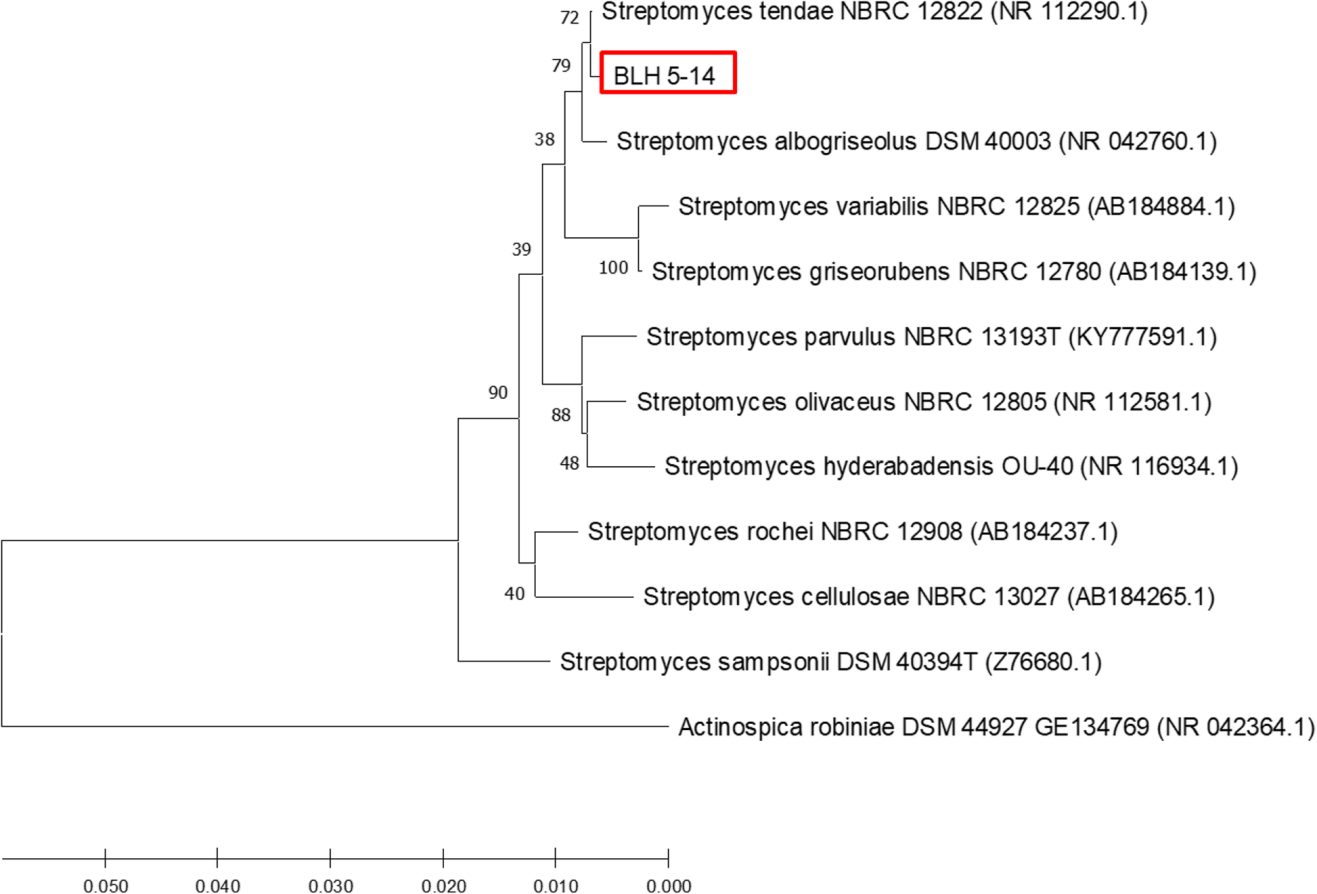
Neighbor-joining phylogenetic tree of genus *Streptomyces* and BLH 5-14 based on 16S rDNA analysis. Bootstrap values based on 1000 replicates are shown at the branch nodes. *Actinospica robiniae* was used as an outgroup

Based on the results of sequence analysis using NCBI BLAST and the obtained phylogenetic tree in Fig. 6, the Actinomycetes isolate (BLH 5-14) belongs to the genus *Streptomyces*, with the closest species being *Streptomyces tendae* strain NBRC 12822 (99.78%).

## Discussion

*Streptomyces tendae* (Ettlinger et al., 1958) was 99.78% similar to BLH-14 isolate. This species was first isolated from soil in Tendae, France. Its characteristics are known to grow in a wide pH range (pH 5.0-12.0) and NaCl concentration of 0-10% [29, 30]. Research by Abdulkhair & Aghuthaymi [31] demonstrated the ability of this species to produce pectinase enzymes and utilize xylose carbon sources.

The production and growth curves obtained from BLH-14 isolates showed similar results with other species of the genus *Streptomyces* in previous studies, namely *Streptomyces coeliflavus* GIAL86 from Meyghan Salt Lake in Iran and *Streptomyces actuosus* A-151 in Taiwan [32, 33]. The optimum day range is generally found from day 5^th^ to day 7^th^, with the highest value obtained at the beginning of the stationary phase. Proteins and enzymes produced in the culture process are known to be in the growth associate group, which will increase and decrease along with the metabolic rate of microorganisms in the culture [34].

The process of characterizing the optimum pH and temperature also showed results that followed previous studies by Kuhad et al. [35] and Nascimento et al. [36]. Both enzymes can remain active at a temperature of 30°C to 70°C (relative activity >50%) and a pH range of 3.0-10.0. These show the potential of BLH 5-14 isolate to be used in the paper bleaching process, replacing compounds such as chlorine and NaOH that can pollute the environment. Pectinase and xylanase enzymes are essential in cutting xylan bonds with polysaccharides, as well as the degradation of pectin on paper during the bleaching process at alkaline pH conditions [37, 38].

The influence of metal ions and chemical compounds on pectinase and xylanase enzymes tends to increase along with the higher concentration of compounds in the reaction solution [39]. Metal ion compounds can form interactions with carboxyl or sulfhydryl groups on proteins, causing disruption of protein structure or helping to increase reaction activity. The inhibitory nature of the Hg^2+^ ion is known to be the result of the interaction with the sulfhydryl group on pectinase and xylanase [40]. Sodium dodecyl sulfate (SDS) acts as a surfactant, causing the denaturation of protein structures along with the disruption of hydrophobic bonds in enzymes [41, 42].

Xylanase enzyme from isolate BLH 5-14 showed decreased activity value due to the administration of the Ethylenediaminetetraacetic acid (EDTA) compound. This result indicates that this enzyme requires metal ions for the reaction process because EDTA acts as a chelating agent which tends to react and attract metal ion compounds in solution [43]. On the other hand, xylanase usually does not respond significantly to the administration of organic alcohol solutions. According to Amobonye et al. [42], this may indicate the presence of a coil-like structure in a higher ratio of protein which tends to be stable in organic solutions, which can be beneficial in industries involving alcohol organic solutions. Some examples include the bioethanol production process, the production of alcoholic beverages, and the process of dissolving non-polar substrates [42, 44, 45].

The analysis of the enzyme molecular size using SDS PAGE and zymograms showed a difference between pectinase and xylanase. Generally, the molecular weight of pectinase from Actinomycetes is in the 35-50 kDa range, with pectate lyase and polygalacturonase types from *Actinomadura keratinilytica* and *Streptomyces coelicolor* [46, 47]. The results of the xylanase molecular size are in the 20-50 kDa molecular weight size range of the *Streptomyces* genus [14]. The presence of the two size enzyme molecules may indicate the presence of 2 types of a xylanase enzyme family by Actinomycetes isolates (BLH 5-14), namely GH11 that generally <30 kDa, and GH10 that >30 kDa [14, 48, 49].

Xylanase enzymes from GH10 and GH11 tend to work synergistically, with GH11 producing large hydrolysis products, and with the help of GH10, can be degraded further into smaller xylan molecules. Results of larger-sized xylooligosaccharide molecules produced as a result of this reaction indicate the endo-type cleavage mechanism [48, 50]. On the other hand, pectinase produces monosaccharide products as the smallest unit, which implies that the pectinase in this study has an Exo type of cleavage. In general, this product can benefit industries with a demand for the direct production of D-galacturonic acid [51, 52]. One example is a dietary supplement in the health industry that can reduce intestinal inflammation and prevent the development of cancer-causing tumor cells [53].

## Conclusion

The results and data from a series of studies show that Actinomycetes isolates from marine sediments of Indonesia identified as *Streptomyces tendae* can produce pectinase and xylanase enzymes. Both show properties susceptible to a wide range of pH and temperature, along with the distinct influence of chemical and metal ion compounds. In the storage process, this characterization procedure can be utilized as a reference, particularly to maintain or even boost the anticipated enzyme activity in large-scale production. In addition to using waste and being cost-effective, oligosaccharide products made from biomass waste, such as galacturonic acid and xylooligosaccharides, are currently a market target due to their numerous applications in the biomedical industry. Several studies regarding in vitro and in vivo assays, as well as purification methods to extract POS and XOS from the fermentation process, have been conducted in previous studies. As a result, there is a greater probability of developing this isolate to produce POS and XOS that will be beneficial and accessible to a larger community.

## Data Availability

The datasets used and analyzed during this research are available from the corresponding author upon reasonable request.

## Abbreviations

POS: Pectic-oligosaccharides
XOS: Xylooligosaccharides
DNS: Dinitrosalicylic acid
OD: Optical density
SDS: Sodium dodecyl sulfate
SDS-PAGE: Sodium dodecyl-sulfate polyacrylamide gel electrophoresis
TLC: Thin layer chromatography
DAP: Diphenylamine acetone phosphoric acid
NCBI: National Centre for Biotechnology Information
BLAST: Basic Local Alignment Search Tool
GH: Glycoside Hydrolases
EDTA: Ethylenediaminetetraacetic acid
